# Neuronal Aneuploidy in Health and Disease: A Cytomic Approach to Understand the Molecular Individuality of Neurons

**DOI:** 10.3390/ijms10041609

**Published:** 2009-04-15

**Authors:** Thomas Arendt, Birgit Mosch, Markus Morawski

**Affiliations:** Paul Flechsig Institute of Brain Research, University of Leipzig, Leipzig, Germany; E-Mail: markus.morawski@medizin.uni-leipzig.de

**Keywords:** *alu*-repeats, Alzheimer's disease, cell cycle, cell death, chromosomal mosaicism, *in situ* hybridisation, laser capture microdissection, neurodegeneration, slide-based cytometry

## Abstract

Structural variation in the human genome is likely to be an important mechanism for neuronal diversity and brain disease. A combination of multiple different forms of aneuploid cells due to loss or gain of whole chromosomes giving rise to cellular diversity at the genomic level have been described in neurons of the normal and diseased adult human brain. Here, we describe recent advances in molecular neuropathology based on the combination of slide-based cytometry with molecular biological techniques that will contribute to the understanding of genetic neuronal heterogeneity in the CNS and its potential impact on Alzheimer's disease and age-related disorders.

## 1. Aneuploidy in the Normal and Diseased Brain

Understanding the mechanisms underlying generation of neuronal variability and complexity remains a basic challange to neuroscience. Structural variation in the human genome is likely to be one important mechanism for neuronal diversity and brain disease. A combination of multiple different forms of aneuploid cells due to loss or gain of whole chromosomes (mosaic aneuploidy) giving rise to cellular diversity at the genomic level have been described in neurons of the normal and diseased adult human brain [1–11]. However, the incidence and regional distribution of neuronal aneuploidy in the human brain, whether it affects all chromosomes to the same extent and its impact on brain development and function still remain obscure.

Aneuploidy is defined as the loss and/or gain of chromosomes giving raise to a numerical deviation from haploid genome multiples [12]. While aneuploid cells have been typically associated with pathophysiological conditions such as cancer [13], Down's syndrome [14], Turner's syndrome [15] and mosaic variegated aneuploidy [16] and idiopathic autism [17], cells in normal individuals have basically been assumed to contain identical euploid genomes [18]. Still, earlier hypotheses suggested that a number of mammalian somatic tissues are populated by polyploid cells. Adult neurons of mammals were assumed to be postmitotic cells characterized to some extent by a polyploid chromosome complement. Testing this hypothesis in the past through histochemical methods, however, yielded controversial results through technical limitations [19,20]. However, with the recent development of molecular cytogenic techniques, aneuploid cells in the normal developing and mature brain have clearly been identified, indicating that the maintenance of aneuploid neurons in the adult CNS is a widespread, if not universal, property of organisation [1–11].

Recent studies of the embryonic brain have shown that about approximately one-third of the dividing cells that give rise to the cerebral cortex have genetic variability, manifested as chromosome aneuploidy [3,7,21]. Neurons that comprise the adult cerebral cortex arise from mitotic neural progenitor cells in the ventricular zone, a proliferating region where aneuploid cells appear to be generated through various chromosome segregation defects initially [7,22]. While a portion of these aneuploid cells apparently die during development [7,23,24], aneuploid neurons have been identified in the mature brain in all areas assayed [3–8,11,25] indicating that aneuploidy does not necessarily impair viability [26]. Aneuploid neurons in the adult have been shown to make distant connections and express markers associated with neural activity which indicates that these neurons can be integrated into brain circuitry [18].

### 1.1. The overall rate of aneuploidy for the full complement of chromosomes of any vertebrate brain remains to be determined

The overall prevalence of aneuploidy in the normal adult mammalien brain is currently unclear [18], and might differ with respect to brain region, type of chromosome complement and species. The percentage of aneuploid neurons in the human cerebellum, for example, is lower than that in the cerebral cortex or olfactory bulb [9], suggesting that there might be inherent differences in the rates of mosaic aneuploidy between brain regions.

Analyses of sex chromosomes in postmitotic cells from mouse cortex and olfactory bulb using chromosome-specific paints indicate that about 1–6% of cells have gained or lost sex chromosomes [3,7,23]. Similar analyses in humans using whole-chromosome paint and locus-specific point probes indicate that about 4% of brain cells, including postmitotic neurons as well as non-neuronal cells, have lost or gained chromosome 21 [7]. A more recent study has even reported an aneuploid rate of 13% for chromosome 21 in hippocampal neurons of the normal adult human brain [27]. In our own studies on chromosome 17 in the entorhinal cortex of the normal adult human brain, we observed an aneuploid rate between 6% and 19% [5]. If these rates of aneuploidy for chromosomes 21 and 17 are indicative of the rates for other chromosomes, the total percentage of aneuploid neurons would represent a considerable quantity in the adult brain. In support of this assumption, Osada *et al*. [25] observed that 64% of normal murine cerebral cortical nuclei have deviations from the euploid karotype following nuclear transfer to oocytes.

A major drawback of these initial studies on aneuploidy in the adult human brain is the fact that for technical reasons, analyses are performed on isolated cellular nuclei. This prevents clear attribution of cytogenetic changes to define subsets of cells and to study these changes in a cytoarchitectonic context of preserved tissue architecture. These technical restrictions make it difficult to study the potential link between cytogenetic cellular individuality and selective neuronal vulnerability which itself follows a defined distribution throughout different brain areas (see below), and, thus, require the implementation of novel techniques. Further, it obscures a clear cut differentiation between neurons as postmitotic cells and glial cells as potentially proliferating cells. Thus, the rate of aneuploidy for the full complement of chromosomes from mouse, human or any other vertebrate brain remains to be determined.

### 1.2. It is unclear what degree of aneuploidy a single cell can support and still survive in the adult brain

When neural progenitor cells are placed into culture with fibroblast growth factor 2 to promote stem cell expansion, a reduction in aneuploidy is observed in the mitotic population. This change can be attributed to a preferential loss of aneuploid cells, indicating their higher susceptibility [7]. Alternatively, however, it could be assumed that aneuploid cells are less well able to proliferate and instead exit the cell cycle and terminally differentiate. A marked decrease in the rate of aneuploidy was also observed during brain development, potentially indicating a higher rate of elimination due to higher susceptibility to cell death. While the rate of aneuploidy in proliferating neuroblasts is as high as 33% [7,21], it decreases to about 10% in the postmitotic cells in the adult brain [5,11,28]. The apoptotic clearance of neuronal cells observed during cortical development [26] might be influenced by inherited mutations in genes implicated in the maintenance of chromosome stability.

Alterations in mechanisms of apoptotic clearance may result in a lack of abnormal neuronal clearance, leading, therefore, to neurodevelopmental abnormalities in childhood. As the onset of neuronal apoptosis is age dependent, the persistence of aneuploidy/polyploidy in the brain throughout postnatal life may be the cause of extensive apoptosis. Because extensive apoptotic clearance should disturb the maintenance of the established neuronal cell content in the adult brain, it may be considered a cause of late-onset neurodegenerative diseases. Different types of chromosomal imbalances may relate to an uneven apoptotic signaling potential. Polyploidy seems to be more significantly involved in the neuronal cell death of postmortem brain samples from AD [10], in contrast to the persistence of aneuploidy in the normal and schizophrenic brain [11,29,30]. Therefore, each type of chromosome complement abnormality might possess a different propensity for apoptotic clearance.

### 1.3. Caution for stem cell therapy

Transplantation of neural stem cells have been suggested as a potential therapeutic tool to treat CNS disorders. As a source of neural stem cells is the developing brain [31], its characteristic features should be reevaluated. The high rate of aneuploidy of mammalian neuronal stem cells, which amounts to about 30% [7,11], however, has not been considered in this context. If, further, organotypic cultivation commonly used to expand the stem cell population induces aneuploidy [11,21], it might cause a considerable risk to use such aneuploid cells that show a high rate of neoplastic transformation [13,32] for therapeutic purposes. The rate of chromosomal abnormalities in human oocytes rages from 10 to 20% [33,34]. The rate of chromosomal mosaicism in preimplantation embryos analyzed at different stages varies widely from 15 to 90%.

### 1.4. The functional significance of a brain composed of an intermixed population of aneuploid and euploid neurons is currently unknown

One possibility is that aneuploidy serves a mechanism for generating cellular diversity within the CNS. Indeed, aneuploid cells display distinct gene expression profiles compared with euploid cells from the same lineage as shown in a mouse model of loss of heterozygosity [3]. This indicates that functional gene expression can be permanently altered in living cells by chromosomal aneuploidy.

As shown recently, moreover, human autosomes typically show a high rate of random monoallelic gene silencing, expressing only either the maternal or the paternal allel [35]. This can clearly lead to differences in expressed protein sequences and to differences in levels of gene expression, a mechanism that further enhances molecular diversity of individual cells. It remains comletely unknown whether similar mechanisms of silencing single allels might be active if due to aneuploidy multiple copies of a single allel are present in a cell.

Through alterations in gene dosage by chromosomal gain or loss, aneuploid cells may also increase susceptibility to disease, as has been suggested for germline mutations resulting in large-scale copy number polymorphisms [36] and locus triplications [37].

Moreover, aneuploidy due to meiotic errors is the most common cause of fetal death, stillbirth, and disorders associated with chromosome abnormalities in humans [38,39]. Apart from trisomy of chromosomes 13, 18, and 21, as well as additional supernumerary marker chromosomes, all remaining autosomal aneuploidy conditions are supposed to occur in liveborns as mosaic forms only. Mental impairment is a characteristic feature of all recognizable autosomal aneuploidy syndromes. It is frequently accompanied by morphological changes in the brain of affected children, supporting the contention that the presence of neuronal cells with a gained or lost autosome may be related not only to impaired functioning but to conspicuous morphological abnormalities [40].

### 1.5. Chromosomal mosaicism confined to the brain might involve different chromosomes relevant to aging and common brain disorders

The two best studied biological processes associated with numerical chromosome imbalances are tumorigenesis and aging. Brain tumors demonstrate aneuploid karyotypes in at least about 50% of cases [41,42]. The variation of chromosome complement observed at the cytogenetic level as a feature of human aging has been well documented [43–45]. More recently, this penomenon was found to be likely explained by mitotic misregulation [46]. Although tissue-specific chromosome complement variation in human aging is poorly understood, current data provide indirect evidence for an acquired aneuploidy in numerous tissues. In addition, the CNS possesses parts in which long-term production of neurons occur and, therefore, low-level chromosomal mosaicism in the human brain may also contribute to aging processes either through mitotic errors or the onset of expression of age-related genes in aneuploid mature neurons.

Human diseases with reported aneuploidy include Alzheimer's disease [7,12,27,47–54], Down's syndrome [55–58], ataxia-telangiectasia [28], schizophrenia [29, 59–64], mosaic variegated aneuploidy [16, 65–68], and various tumors such as gliomas, glioblastomas and medulloblastomas [41,69,70–72].

Early studies on AD have proposed a link between aneuploidy, particular of chromosome 21, and AD. This suggestion was based on two observations. First, the gene coding for APP which through abnormal processing gives rise to Aß being critically involved in AD pathology, is located on chromosome 21. Second, Down's patients, trisomic for chromosme 21, frequently develop an AD-type pathology at higher age. Subsequent studies on gene dosage of chromosome 21 gene in AD, however, have reported conflicting results and the actual role of gene dosage through trisomy 21 in AD remains controversial. Prior studies using allele-specific PCR on sporadic and familial AD cases have failed to observe dosage increase [73–75]. Notably, these PCR approaches might have lacked the sensitivity to detect increased mosaic levels of trisomy compared to controls. In addition, increased trisomy could be masked by the possibility that neural trisomy shows spatial variation, combined with the known existence of chromosome 21 monosomy. Furthermore, a recent study of five families with autosomal dominant early-onset AD and hereditary dementia with cerebral amyloid angiopathy showed duplication of the APP locus on chromosome 21, as verified by quantitative multiplex PCR and dual FISH [76].

Whether there are significant levels of trisomy 21 in the brains of AD patients is still unknown. The longevity of postmitotic neurons might provide more time for the accumulation of aneuploidy, in contrast to more rapidly cycling populations. This suggestion is supported by the observation of significant levels of chromosome 21 monosomy and trisomy in normal human neural tissue compared to lymphocytes [8].

Thus, future studies will be neccessary to directly address the question whether AD is characterised by increased aneuploidy of one particular chromosome (e.g. 21) or perhaps by multiple chromosomes as in diseases like schizophrenia or cancer [29].

## 2. Selective Vulnerability of Cortical Neurons Reflect the Modular Cortical Structure

Neurodegenerative changes in AD are not randomly distributed throughout the cerebral cortex but rather follow a defined pattern of progression that provides the basis for the neuropathological staging [77]. Both, certain cortical areas and types of neurons are earlier and more constanly involved than others. Neurofibrillary degeneration in the cerebral cortex preferentially affects lamina III and V pyramidal neurons, leaving interneurons unaffected. Further, degeneration preferentially affects the limbic cortex and cortical association areas while primary sensory and motor areas are largely spared. The reasons for this selective neuronal vulnerability are unkown.

Neurofibrillary degeneration in the cerebral cortex, moreover, typically occurs in clusters. Degeneration predominantly involves pyramidal neurons within supra- and infragranular layers with the clustering in these layers often being in register with each other [78] indicating a columnar organisation of degeneration [79]. This NFT clustering is highly correlated with symptoms [80]. Also plaques appear to show a degree of vertical clustering between apical dendritic clusters [81].

The columnar structure is disorganised in cortical areas affected by pathology in AD [82]. Cortical atrophy in AD, moreover, is due to a decrease in cortical length indicating a shrinkage or loss of columns while cortical thickness remains unaffected [83,84]. Also during normal aging, minicolumn width shinks in those cortical regions that are potentially vulnerable to degeneration in AD [85]. A study on trisomy 21 Down's patients, furthermore, suggest that children with the disorder exhibit early adult-like neuronal mini-column spacing [86]. This may relate to the apparently accelerated aging and early onset of AD in Down's syndrome.

This issue of columnar organisation and clustering has been pursued with respect to other pathological structures. Disruption of the columnar organisation of glial processes has been reported for AD [87]. Lewy bodies and Pick bodies [88,89] also exhibit clustered distribution which reflect a modular structure. Taken together, available evidence on the distribution of cortical pathology indicates a determination of the pattern of pathology through the modular organisation of the cortex [90] which basically is a structural reflection of its ontogeny.

## 3. Ontogenetic and Phylogenetic Aspects of Brain Pathology

AD is a disorder that selectively affects the human brain. It has been suggested that AD is specific to man because of molecular genetic events which promoted a rapid evolution of the hominid brain [91,92]. The phylogenetic expansion of the hominid neocortex most likely arose through a rapid multiplication of the fundamental columnar building blocks.

During embryonic development of the cerebral cortex, cells migrate towards the surface and form mini-columns of cells. They appear to be grouped into larger macro-columns, which form the basis of the mapping of functions across the brain's surface.

Pyramidal neurons, potentially vulnerable to neurofibrillary degeneration in AD, according to the radial unit hypothesis, derive from radially migrating neurons that originate in the ventricular zone of the pallium (cortex). In the primate brain, they find their way to the distant cortex by using radially oriented glial fibres as guides. On the contrary, cortical interneurons, unaffected by degeneration in AD, are born outside the cortex in the ganglionic eminence and reach their final position through tangential migration [93,94].

Following the last mitotic division, immature neurons of the ventricular and sub-ventricular zones attach to an adjacent set of glial guidance fibres. Thus, clonally related neurons generated serially in time at the same locus in the germinal epithelium migrate sequentially along the same or adjacent set of glial guidance fibres, and settle in the inside to outside pattern aligned in a single vertical column [95–97]. Neurons of this radial column form an ontogenetic unit, the fundamental building block in the developing neocortex [98]. Thus, the basic columnar organisation of the cortex reflects its mode of generation [99–101].

The evolutionary expansion of neocortical size in mammals which is particularly prominent in anthropoid primates (i.e. monkeys, apaes, humans) reflects an increased number of cortical cells. According to the radial unit hypothesis, this phylogenetic expansion in cortical surface area results from an increase in the number of founder cells prior to neurogenesis. This increase in the number of cells is generated by two mechanisms: (i) an increase in the size of the embryonic subventricular zone and (ii) a prolonged period of cell cycle activity of progenitor cells during neurogenesis [102]. In macaque monkey, the period during which cell division occurs is 10 times longer than in rodent and the cell cycle duration is two to five times longer than in mouse [103]. Substantially more total rounds of cell divisions elapse during the prolonged neurogenetic period of the monkey cortex, providing the basis for increased cell proliferation. Moreover, unlike the progressive slowing that occurs during cortical development in rodents, cell division accelerates during neurogenesis of the enlarged cortical layers in monkeys. These findings suggest that evolutionary modifications of the duration and number of progenitor cell divisions contribute to both the expansion and laminar elaboration of the primate neocortex [104]. The enlarged cortex of great apes reflects a longer period of neuronal formation during pre-natal development, so that each dividing progenitor cell undergoes more cell cycles before stopping cell division [104]. Cortical progenitors undergo 11 rounds of cell division in mice [105], at least 28 in the macaque [104], and probable four more in human [106]. This phylogenetic prolongation of mitotic activity provides the basis for a potentially higher rate of accumulating mitotic errors during neurogenesis. It is unclear, however, whether different mammalian species differ with respect to the frequency of aneuploid neurons in the CNS.

## 4. A Cytomic Approach to Study Molecular Brain Architecture

Until recently, the DNA amounts of defined single neurons have been difficult to study under conditions of preserved tissue architecture. Although chromosome mapping on interphase nuclei by fluorescence *in situ* hybridisation (FISH) has made considerable progress in recent years [4,7,21,107], it still suffers from major technical limitations. These limitations mainly arise from the application of FISH to isolated cellular nuclei which prevents from clearly attributing cytogenetic changes to defined subsets of cells and to study these changes in a cytoarchitectonic context of preserved tissue architecture. These technical restrictions make it difficult to study the potential link between cytogenetic individuality and selective neuronal vulnerability and, thus, require the implementation of novel techniques.

Therefore, we have developed a novel approach for the DNA quantification of single identified neurons in brain slices based on a combination of slide-based cytometry (SBC) after DNA staining with propidiumiodide, chromogenic *in situ* hybridisation (CISH) with chromosome-specific probes and laser microdissection followed by quantitative PCR (qPCR) of *alu* repeats [5,108]. All three methods are applicable to tissue sections and can be combined with immunocytochemical detection of specific marker proteins which allow for further specification of cellular identity in the context of preserved tissue architecture. This is a clear advantage when analysing tissue sections containing different cell types (e.g. neurons versus glia) and modes of pathological affections (e.g. cytoplasmic inclusions, neurofibrillary degeneration).

**Figure 1.**
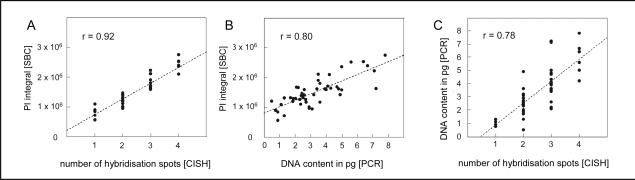
Inter-method reliability of three independent methods for single cell DNA quantification. A set of 48 microscopically identified neurons of the entorhinal cortex in a patient with early AD was evaluated through subsequent application of SBC, CISH and PCR amplification of *alu* repeats. Tissue sections were first processed for SBC, followed by hybridization with a chromosome 17-specific probe. Subsequently, indentified neurons were captured through laser microdissection and subjected to PCR amplification of *alu* repeats. Regression analyses reveal correlation coefficients according to Bravais-Pearson of (A) r = 0.92 for the SBC data versus hybridization results (CISH), (B) of r = 0.80 for LSC versus PCR amplification and (C) r = 0.78 for PCR amplification versus hybridization. All correlation coefficients are significant at p < 0.001 (adapted from [5]).

To confirm our experimental approach we cross validated the three methods, i.e SBC, CISH and qPCR through subsequent application one by one to a defined subset of microscopically identified neurons and obtained a remarkably high inter-method reliability (Figure 1). Obviously, this combination of the three methods in a row can be easily adapted to other issues. When the PI intergral (SBC) is plotted against the number of hybridisation spots (CISH), a highly significant correlation will be obtained (Figure 1A). Similarly, the PI integral shows a highly significant correlation with the DNA content determined by PCR amplification of *alu* repeats (Figure 1B). The DNA amount determined by PCR amplification of *alu* repeats also correlates highly significantly with the number of hybridisation spots (Figure 1C).

Based on these correlations, the DNA amount (mean ± SD) of a single diploid neuron identified by two hybridisation spots can be determined as 2.52 ± 0.87 pg, while the DNA content of a neuron with four spots amounts to 5.94 ± 1.28 pg. For comparison, the mean single cell DNA content (±SD) of lymphocytes treated identically to brain tissue and used for control experiments was determined at 2.07 pg ± 0.6 pg.

As in our studies the DNA content of single neurons determined by SBC is far better correlated to CISH (Figure 1A) than is the qPCR measurement (Figures 1B/C), we conclude that SBC is a very reliable technique for future studies on the single cell DNA content in tissue sections.

### 4.1. Quantification of single neuron DNA amount by Slide-based Cytometry (SBC)

**Figure 2.**
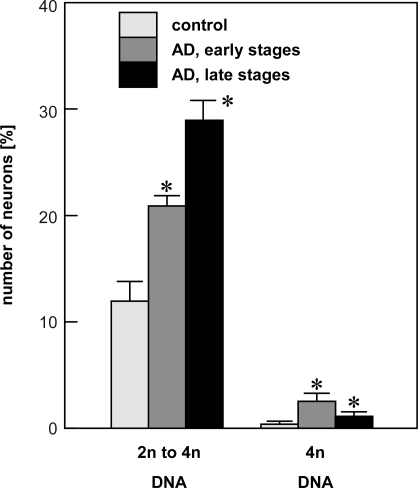
Quantification of single-neuron DNA content by SBC. Note the increase in aneuploid neurons in AD. (control: n=13; AD early stages, Braak stage I/II: n=6; AD late stages, Braak stage V/VI: n=7; * p<0.01; adapted from [5].

For cytometric quantification of DNA, neurons of the entorhinal cortex were identified by immunocytochemical detection with the pan-neuronal neurofilament marker SMI311 and counter-reacted with PI [5]. DNA analysis by SBC identified the majority of neurons in controls as diploid. In our study, we identified about 88% of neurons in control brains with a DNA amount of 2n, while about 12% had a hyperploid DNA content between 2n (diploid) and 4n (tetraploid). About 0.4% of neurons contained a tetrasomic DNA content (Figure 2). In AD, a significant shift of neurons from the diploid to hyperploid and tetraploid DNA content was observed. Neurons with a diploid DNA content were reduced to 77% and 70 % in early and advanced AD, respectively, while the fraction of neurons containing DNA between 2n and 4n was increased to 21% and 29%. The frequency of neurons with a tetrasomic content of DNA was increased in early AD by factor six (2.4%) and more than doubled in advanced AD (1.1%).

### 4.2. Chromogenic in situ hybridization

**Figure 3.**
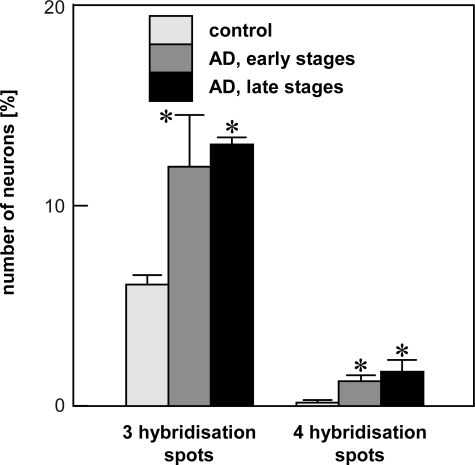
Quantification of CISH signals (chromosome 17 probe) in neurons of the entorhinal cortex (For number of cases compare Figure 2; *p<0.01; adapted from [5]).

After chromogenic *in situ* hybridisation (CISH) with a chromosome 17 probe on brain sections, two hybridisation spots were obtained for the majority of neurons in the entorhinal cortex of both controls and AD [5]. In addition, neurons with none, one or three spots were observed. A small but constant number of neurons in both controls and AD displayed four hybridisation spots. The number of nuclei with three hybridisation spots was doubled in AD compared to controls (controls: 6%; AD early: 12%; AD advanced: 13%). In controls, a small portion of about 0.3 % of neurons showed four hybridisation spots. The relative number of these neurons with four spots was increased in AD by factor four to five (early AD: 1.3%; advanced AD: 1.5%) (Figure 3).

### 4.3. Laser capture microdissection of identified neurons and PCR amplification of alu-repeats

**Figure 4.**
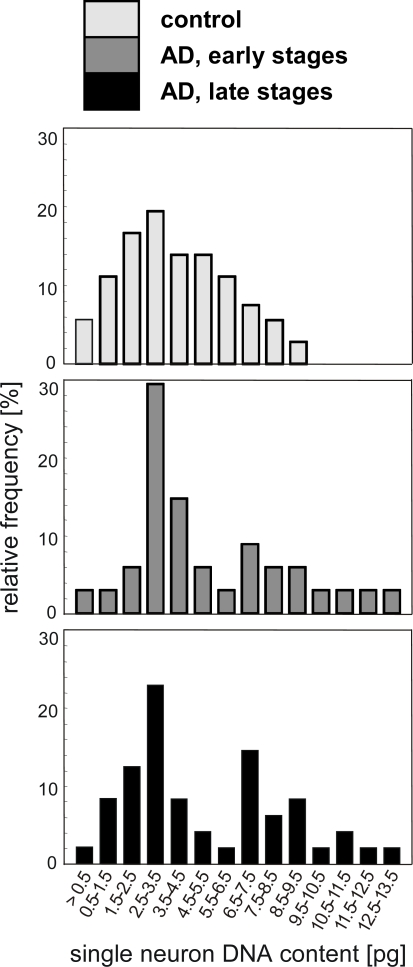
Frequency distribution of single neuron DNA-content determined by PCR amplification of *alu* repeats. Note in AD the shift towards higher DNA content and the appearance of a second peak corresponding to a tetraploid DNA content (adapted from [5]).

Subsequently to CISH, we further analysed the single cell DNA content on the same sections by laser capture microdissection of neurons individually identified under the microscope and subsequent PCR amplification of *alu* repeats [5]. The frequency distribution of single cell DNA content obtained by this method is displayed in Figure 4. Comparing AD to controls, a shift towards higher size classes and differences in the shape of the distribution becomes apparent. The distributions of control groups have a single maximum at 2.5–3.5 pg per cell which corresponds to the size for a 2n DNA content as determined in initial validation experiments (see above and Figure 1). In addition, AD groups displayed a second maximum in the size group of 6.5–7.5 pg per cell most likely representing tetraploid neurons.

## 5. Conclusions

The results of the present study on the DNA amount of identified cortical neurons, obtained through a combination of slide-based cytometry, chromogenic *in situ* hybridization and PCR amplification of *alu-*repeats, indicate that at least two different mechanisms need to be distinguished giving rise to a tetraploid DNA content in the adult brain. Constitutional aneuploidy in differentiated neurons of the normal human brain might be more frequent than previously thought. It is, however, not elevated in AD. In addition, in AD some neurons have re-entered the cell cycle and entirely passed through a functional interphase with a complete DNA replication. The combination of cytometry with molecular biological characterisation of single microscopically identified neurons as outlined here might be a promising approach to study molecular neuronal individuality in the context of preserved tissue architecture. It reflects the concept of cytomics and will forward our understanding of the molecular architecture and functionality of neuronal systems [109,110].
